# Neonatal Cholestasis Associated With Transient Congenital Hyperinsulinism: A Case Report

**DOI:** 10.7759/cureus.80425

**Published:** 2025-03-11

**Authors:** Kyosuke Ibi, Yoshihiko Shitara, Natsuho Adachi, Hiroyuki Tanaka, Naoto Takahashi

**Affiliations:** 1 Pediatrics, The University of Tokyo Hospital, Tokyo, JPN

**Keywords:** biliary atresia, cholestasis, congenital hyperinsulinism, diazoxide, fetal distress, hypoglycemia, jaundice, neonate

## Abstract

Congenital hyperinsulinism (CHI) and neonatal cholestasis (NC) are occasionally observed in neonatal settings; however, the causes have not been identified despite thorough investigation. Surgical evaluation is essential for patients with cholestasis to rule out biliary atresia because timely surgical intervention is critical. Few case reports have described the co-occurrence of CHI and NC. Herein, we report a case of a boy born as one of dichorionic-diamniotic twins via planned cesarean delivery. Apparent ischemic events were not present, with Apgar scores of 8 and 9 at one and five minutes, respectively; however, the umbilical artery blood gas pH was 7.17. He experienced a hypoglycemic attack on day two and was diagnosed with CHI. The patient was put on diazoxide therapy, following which his blood glucose levels were stable without intravenous glucose infusion. However, cholestasis was observed from day 14. Workups did not indicate any specific clinical condition, and biliary atresia was ruled out on day 44 by cholangiography. He exhibited prolonged fasting hypoglycemia because of lipid malabsorption until he recovered from cholestasis. He was free of diazoxide at 16 months of age and subsequently acquired normal physical and mental development. This case highlights the potential co-occurrence of CHI and NC. The etiologies remain unidentified; however, both may have resulted from perinatal distress. Hypoglycemic episodes prior to the diagnosis of CHI may also trigger cholestasis. Transient CHI can be accompanied by benign NC, and such patients can experience persistent hypoglycemia and require nutritional management until the cholestasis is relieved.

## Introduction

Congenital hyperinsulinism (CHI) is the leading cause of recurrent hypoglycemia in infants and is observed in 1/50,000 births [1]. CHI is commonly classified as either transient or permanent. Transient CHI can occur owing to certain risk factors, including birth asphyxia, intrauterine growth retardation, and maternal diabetes mellitus, or associated with various overgrowth syndromes, such as Beckwith-Wiedemann syndrome or metabolic conditions such as congenital disorders of glycosylation [2]. CHI associated with genetic mutations has also been described [1]. Neonatal cholestasis (NC) is an uncommon but potentially serious condition linked with hepatobiliary dysfunction. The incidence of NC is estimated to be approximately one in 2,500 live births [3]. The etiologic categories include both extrahepatic and intrahepatic disorders, including biliary atresia (BA), neonatal infections, genetic and inborn errors of metabolism, endocrine disorders, toxin and drug exposures, hypoxia/ischemia, idiopathic neonatal hepatitis (now referred to as transient neonatal cholestasis), and other miscellaneous causes [4,5].

In the clinical setting, the etiologies of both conditions are often unknown, as they refer to a heterogeneous group of disorders. Herein, we present a case of a preterm infant diagnosed with NC during treatment for CHI. This report aims to highlight the co-occurrence of these two conditions and their possible etiologies.

## Case presentation

The patient was a boy born at 36 2/7 weeks’ gestation with a birth length of 45.5 cm (-0.46 standard deviation (SD)) and birth weight of 2,364 grams (-0.68 SD) as one of dichorionic-diamniotic twins by planned cesarean delivery to a 33-year-old mother. This was her second pregnancy conceived through artificial insemination with the husband’s semen, and the first child was four years old with an unremarkable medical history. The mother had no history of gestational diabetes mellitus but was taking propylthiouracil for Graves’ disease. The mother remained in a euthyroid state throughout pregnancy. The patient’s Apgar scores were 8 and 9 at one and five minutes, respectively, and the umbilical artery blood gas pH was 7.17. The patient’s twin was born 48.0 cm (+0.70 SD) in length and 2,990 g (+1.21 SD) in weight, with Apgar scores of 8 and 9 at one and five minutes, respectively, and umbilical artery blood gas pH of 7.26. The patient was initiated on oral feeding as routine management. On day two, the patient developed apnea and seizures due to low blood glucose levels (below the detection limit) and was administered an intravenous glucose infusion. The blood insulin level in the critical sample was disproportionately high (3.9 µg/dL), and the infant required a high glucose infusion rate (maximum 16.6 mg/kg/min on day two) to maintain normal blood glucose levels. The patient was diagnosed with CHI, and since he could not maintain blood glucose levels without intravenous glucose infusion, diazoxide (5.0 mg/kg/day) was introduced on day seven, and the dose was steadily increased. Diazoxide was highly effective, leading to the discontinuation of intravenous glucose infusion on day 11. However, on day 14, the patient had pale stools and direct hyperbilirubinemia. He was then referred to our hospital on day 27 for a detailed investigation, with BA as a differential diagnosis. The patient’s sibling was discharged without any medical complications. Upon admission, the patient presented with liver damage and cholestasis, with aspartate aminotransferase, alanine aminotransferase, direct bilirubin, and total bile acid levels measuring 183 U/L, 66 U/L, 4.0 mg/dL, and 133.6 µmol/L, respectively. α-fetoprotein levels were disproportionately elevated for the patient’s age [6-8]. Complete blood cell count parameters were within normal range (Table 1).

**Table 1:** Laboratory tests on admission (day 27).

Parameters	Value	Reference range
Complete blood count		
White-cell count (per μL)	10,400	3,300–8,600
Hemoglobin (g/dL)	13.8	13.7–16.8
Hematocrit (%)	41	40.7–50.1
Platelet count (per μL)	477,000	158,000–348,000
Serum chemistry		
C-reactive protein (mg/dL)	0.07	0.0–0.3
Total protein (g/dL)	5.1	6.6–8.1
Albumin (g/dL)	3.6	4.1–5.1
Aspartate aminotransferase (U/L)	183	13–30
Alanine aminotransferase (U/L)	66	10–42
γ-glutamyl transpeptidase (U/L)	271	13–64
Alkaline phosphatase (U/L)	529	38–113
Total bilirubin (mg/dL)	9.0	0.4–1.5
Direct bilirubin (mg/dL)	4.0	0.0–0.3
Total bile acid (μmol/L)	133.6	0.0–14.4
α-fetoprotein (ng/mL)	76,603	0.0–9.0
Urea nitrogen (mg/dL)	2.5	8–20
Creatinine (mg/dL)	0.17	0.65–1.07

Parental nutrition-associated cholestasis was unlikely, as the patient required intravenous glucose infusion for only nine days. No congenital or acquired infectious diseases were detected during the diagnostic workup. He did not have acidemia or hyperammonemia. Metabolic disorders, including neonatal intrahepatic cholestasis caused by citrin deficiency, were not identified during newborn screening, plasma amino acid analysis, or organic acid testing. Echocardiography revealed a normal heart structure. Blood tests were normal for pituitary function, and no abnormalities were found on head magnetic resonance imaging. An abdominal ultrasound revealed a normal-sized liver without any space-occupying lesions. The gallbladder appeared small and contracted, showing no contractility on pre- and post-prandial ultrasound films. Hepatobiliary scintigraphy performed on day 30 demonstrated no bowel excretion, even in 24-hour delayed images. Duodenal fluid examination on day 32 detected trace amounts of bilirubin and total bile acids. The possibility of BA could not be fully ruled out based on the above examinations. Liver damage and cholestasis were not relieved by medication. On day 44, the patient underwent cholangiography during laparotomy, and BA was ultimately ruled out. A liver biopsy performed during laparotomy showed nonspecific findings of prominent cholestasis with hepatocyte degenerative changes and ductular reaction. Nutritional management and medication were continued, including the administration of ursodeoxycholic acid and supplementation with fat-soluble vitamins. Blood glucose levels were controlled by increasing the diazoxide dose to 15 mg/kg/day on day 56. Hydrochlorothiazide (1 mg/kg/day) was given from days 56 to 105 to prevent fluid retention owing to the side effects of diazoxide, and the patient did not encounter any adverse effects from diazoxide.

**Figure 1:**
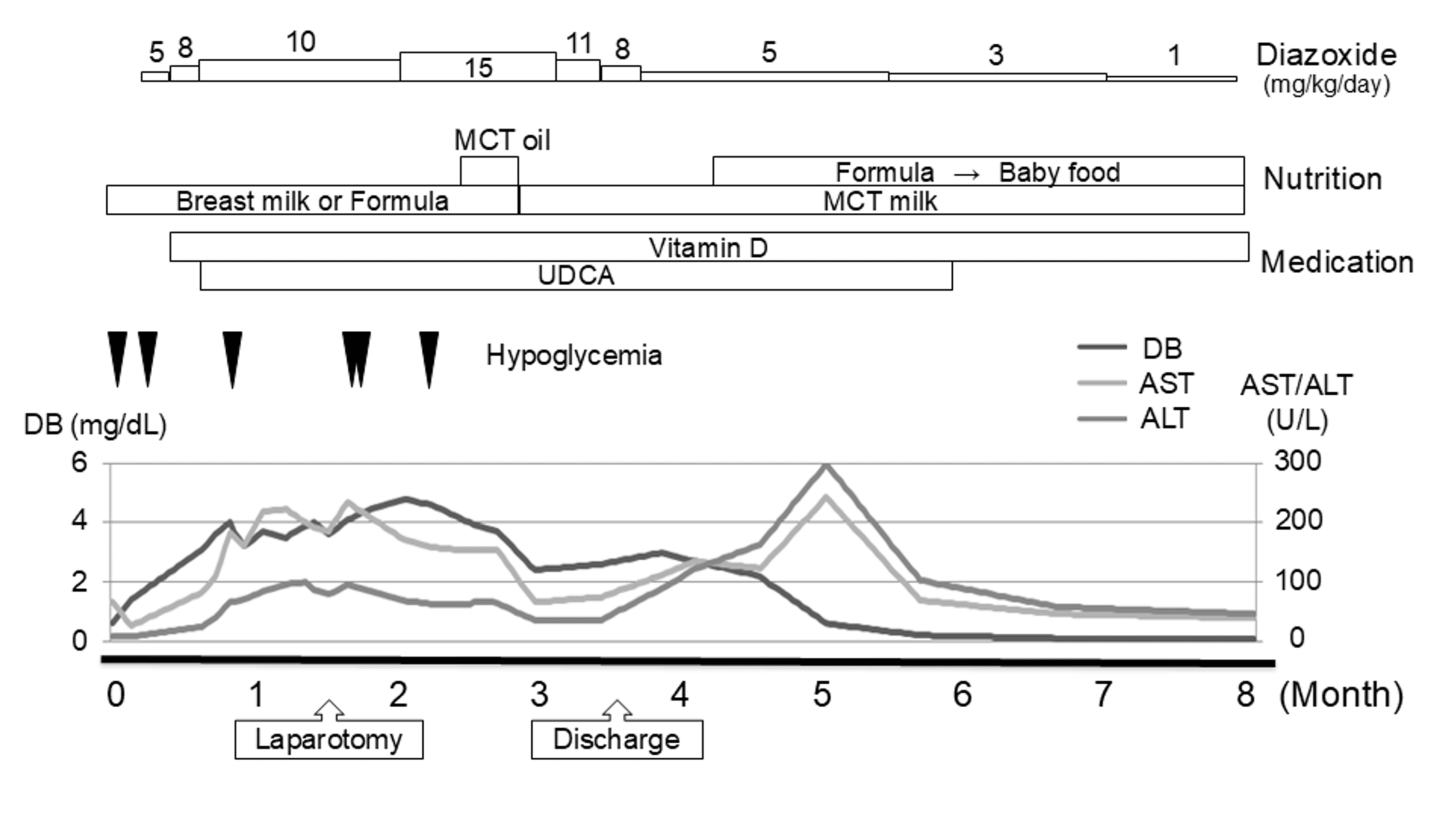
Summary of the patient’s clinical course until cholestasis remission. The patient experienced multiple courses of hypoglycemic episodes (blood glucose < 45 mg/dL). Cholestasis (indicated by aspartate aminotransferase (AST), alanine aminotransferase (ALT), and direct bilirubin (DB) levels) was stabilized by medication with ursodeoxycholic acid (UDCA) and fat-soluble vitamins. Nutritional management included the use of medium-chain triglyceride (MCT) oil and milk.

Although we assumed that the patient had transient CHI, he experienced hypoglycemia (fasting blood glucose < 45 mg/dL [9]) when we attempted to allow a feeding interval (28 mg/dL on day 28, 43 mg/dL on day 48, and 37 mg/dL on day 51). On day 62, blood samples were collected, and his blood glucose level was found to be 20 mg/dL, revealing that he no longer had hyperinsulinemia (blood insulin level < 1.0 µg/dL). We suspected that impaired lipid absorption due to cholestasis was an etiology of prolonged hyperglycemia other than transient CHI. Medium-chain triglyceride (MCT) oil was initiated on day 65 and was switched to MCT milk on day 81. He maintained a stable blood glucose level even with an extended feeding interval after cholestasis had passed its peak. We decreased the dose of diazoxide to 7 mg/kg/day and confirmed that he could maintain normal blood glucose levels. He was discharged on day 108. Liver damage, cholestasis, and α-fetoprotein levels gradually subsided each month. Ursodeoxycholic acid was discontinued when the patient reached five months of age. Diazoxide-induced liver injury was not highly indicated because cholestasis spontaneously disappeared during diazoxide use. We resumed formula milk at four months of age, started oral food at six months old, and discontinued MCT milk at 10 months of age (Figure 1).

The patient was diazoxide-free when he was 16 months old, with stable blood glucose levels. He had no episodes of hypoglycemia during standard meals, maintained normal liver function, and acquired normal physical and mental development during follow-up. We used the Kyoto Scale of Psychological Development 2020, a standardized developmental assessment in Japan that covers postural-motor (P-M), cognitive-adaptive (C-A), and language-social (L-S) functions at 18 months of corrected age [10]. His developmental quotient was 100, with the P-M domain being 104, C-A being 103, and L-S being 88. At the latest follow-up at 24 months of chronological age, the child measured 83.1 cm (-0.78 SD) in height and weighed 13.1 kg (+1.23 SD).

## Discussion

Herein, we report a combination of transient CHI and benign NC during the neonatal period. Diazoxide was highly effective; however, hypoglycemic episodes followed a protracted course due to cholestasis. Both the CHI and NC were relieved during the infantile period. Subsequently, the patient exhibited normal physical and mental development.

CHI and NC are occasionally observed in neonatal settings and often resolve spontaneously. A striking feature of this case report is the combination of CHI and NC. No apparent etiology was observed in our patient. Genetic analysis for CHI was postponed because the patient was responsive toward diazoxide [2,11].

There have been few reports on the combination of CHI and NC, both of which have unknown causes. In a retrospective study by Jacquemin et al., 10/92 (10.9%) neonates with cholestasis experienced hypoglycemia before cholestasis [12]. Edwards et al. reported that 30 of 63 patients with CHI (47.6%) presented with NC without any apparent cause, except for one patient with galactosemia [13]. Fetal distress and lower birth weight z-scores have been reported to be associated with the development of conjugated hyperbilirubinemia. However, they did not report the low blood glucose levels in patients with and without cholestasis. The reason for the co-occurrence of CHI and NC has not yet been confirmed; however, both transient CHI and benign NC are occasionally associated with fetal and neonatal stress. Perinatal stress is associated with hepatic hypoxia or ischemia [12,14] and hyperinsulinemic hypoglycemia, which may persist for several weeks postnatally [14]. Additionally, hypoglycemic episodes themselves contribute to perinatal stress. Thus, perinatal stress may be a confounding factor linking CHI and NC. In the present case, the patient may have experienced substantial fetal stress as part of the twins, considering the low pH of the umbilical artery blood gas. The twin brother, who showed no evidence of fetal distress, presented with neither CHI nor NC.

We managed the prolonged fasting hypoglycemia in the present case. The patient was not in a hyperinsulinemic state when hypoglycemia was observed on day 62. We suspected that the patient had transient CHI and that there was another reason for persistent hypoglycemia. We concluded that impaired lipid absorption caused by cholestasis contributed to persistent hypoglycemia, which was effectively prevented by nutritional management with MCT milk. His blood glucose levels stabilized after the cholestasis disappeared. Notably, prolonged fasting hypoglycemia can be observed in patients with a combination of CHI and NC. However, they can expect spontaneous remission under both conditions and good physical and mental development.

## Conclusions

The reasons for NC are occasionally not apparent, and a timely surgical workup for BA is indispensable in infants with cholestasis. Transient CHI can be accompanied by benign NC, both of which may result from perinatal distress. Hypoglycemia may also contribute to the development of cholestasis. Patients with transient CHI accompanied by NC can experience persistent hypoglycemia and require nutritional management until the cholestasis is relieved.

## References

[1] Krawczyk S, Urbanska K, Biel N (2022). Congenital hyperinsulinaemic hypoglycaemia—a review and case presentation. J Clin Med.

[2] Demirbilek H, Hussain K (2017). Congenital hyperinsulinism: diagnosis and treatment update. J Clin Res Pediatr Endocrinol.

[3] Suchy FJ (2004). Neonatal cholestasis. Pediatr Rev.

[4] Fawaz R, Baumann U, Ekong U (2017). Guideline for the evaluation of cholestatic jaundice in infants: joint recommendations of the North American Society for Pediatric Gastroenterology, Hepatology, and Nutrition and the European Society for Pediatric Gastroenterology, Hepatology, and Nutrition. J Pediatr Gastroenterol Nutr.

[5] Feldman AG, Sokol RJ (2020). Recent developments in diagnostics and treatment of neonatal cholestasis. Semin Pediatr Surg.

[6] Lee PI, Chang MH, Chen DS, Lee CY (1989). Serum alpha-fetoprotein levels in normal infants: a reappraisal of regression analysis and sex difference. J Pediatr Gastroenterol Nutr.

[7] Mizejewski GJ (2003). Levels of alpha-fetoprotein during pregnancy and early infancy in normal and disease states. Obstet Gynecol Surv.

[8] Lee PI, Chang MH, Chen DS, Hsu HC, Lee CY (1990). Prognostic implications of serum alpha-fetoprotein levels in neonatal hepatitis. J Pediatr Gastroenterol Nutr.

[9] Thornton PS, Stanley CA, De Leon DD (2015). Recommendations from the Pediatric Endocrine Society for evaluation and management of persistent hypoglycemia in neonates, infants, and children. J Pediatr.

[10] Koyama T, Osada H, Tsujii H, Kurita H (2009). Utility of the Kyoto Scale of Psychological Development in cognitive assessment of children with pervasive developmental disorders. Psychiatry Clin Neurosci.

[11] Takasawa K, Iemura R, Orimoto R (2024). Clinical management of diazoxide-unresponsive congenital hyperinsulinism: a single-center experience. Clin Pediatr Endocrinol.

[12] Jacquemin E, Lykavieris P, Chaoui N, Hadchouel M, Bernard O (1998). Transient neonatal cholestasis: origin and outcome. J Pediatr.

[13] Edwards M, Falzone N, Harrington J (2021). Conjugated hyperbilirubinemia among infants with hyperinsulinemic hypoglycemia. Eur J Pediatr.

[14] Stanley CA, Rozance PJ, Thornton PS (2015). Re-evaluating "transitional neonatal hypoglycemia": mechanism and implications for management. J Pediatr.

